# Injury patterns following simple elbow dislocation: radiological analysis implies existence of a pure valgus dislocation mechanism

**DOI:** 10.1007/s00402-020-03541-0

**Published:** 2020-08-11

**Authors:** Marc Schnetzke, Alexander Ellwein, Dirk Maier, Ferdinand Christian Wagner, Paul-Alfred Grützner, Thorsten Guehring

**Affiliations:** 1 BG Trauma Center Ludwigshafen at the University of Heidelberg, Clinic for Trauma and Orthopaedic Surgery, Ludwigshafen on the Rhine, Germany; 2 Department for Orthopaedic Surgery, Medical School Hannover, DIAKOVERE Annastift, Anna-von-Borries-Straße 1-7, 30625 Hannover, Germany; 3 Faculty of Medicine Medical Center, Albert-Ludwigs-University of Freiburg, Hugstetter Strasse 55, 79106 Freiburg, Germany; 4 Shoulder and Elbow Surgery, Arcus Sportklinik ARCUS Kliniken, Rastatter Str. 17-19, 75179 Pforzheim, Germany; 5 ATOS Clinic Heidelberg, German Joint Center Heidelberg, Heidelberg, Germany

**Keywords:** MRI, Posterolateral, Posteromedial, Lateral collateral ligament, Medial collateral ligament, Ulnohumeral joint, Instability

## Abstract

**Introduction:**

The aim of the present study was to analyze the injury pattern and thus the dislocation mechanism after simple elbow dislocation using radiographs and magnetic resonance imaging (MRI) data sets.

**Materials and methods:**

The MRI data sets of 64 patients with a mean age of 44 years (18–77 years) were analyzed retrospectively. The inclusion criteria for the study were (1) radiograph with confirmed simple elbow dislocation, (2) low-energy trauma, (3) MRI of the affected elbow ≤ 3 weeks after trauma. The dislocation direction was determined using radiographs. The integrity of the lateral collateral ligament complex (LCLC), common extensor origin (CEO), anterior capsule (AC), medial collateral ligament (MCL), and common flexor origin (CFO) as well as the joint congruity were assessed based on MRI.

**Results:**

34 patients (53%) had a posterolateral, 26 patients (41%) a posterior, and 4 patients (6%) a posteromedial dislocation. LCLC and AC were affected in 64 out of 64 patients (100%). MCL was affected in 58 patients (91%). CEO were affected in 25 patients (39%) and the CFO in 20 patients (31%). In 11 patients (17%) the injury pattern was more pronounced medially than laterally (MCL, CFO, LCLC), with 2 of these patients exhibiting only a partial LCLC tear. All cases with joint incongruency (*n* = 12, 19%) showed CEO and/or CFO involvement.

**Conclusions:**

Simple elbow dislocation leads to a very heterogeneous spectrum of soft tissue injury pattern. A small proportion of patients showed medially pronounced injury patterns. These findings strongly indicate existence of a “reversed Horii circle” with an underlying valgus mechanism (medial force induction) originating and continuing from medial to anterior.

## Introduction

The so called “simple” (or ligamentous) elbow dislocation is defined as a dislocation of the ulnohumeral joint with a purely soft tissue injury pattern [9]. Analysis of the injury mechanism and the resulting injury pattern is essential for an understanding of this injury and also for treatment planning [1, 5, 6, 19, 20]. The combination of clinical findings (e.g. joint gap widening under stress test fluoroscopy) and affirmation of signs of instability in the MRI (e.g. drop sign, joint incongruency) lead to further treatment selection. As early as 1992, O'Driscoll et al. studied the accident process in detail: according to this, a supination moment coupled with valgus stress results in rupture of the lateral collateral ligament complex and the posterolateral parts of the capsule and hence in dislocation [15]. On this basis, Horii and O ‘Driscoll et al. postulated the “Horii circle of soft tissue injury”. This circle theory states that the soft tissue injury proceeds sequentially from lateral to medial. In contrast, there are other alternative approaches that reconstruct the injury process [12, 16, 22, 23]. Scheiber et al. evaluated video sequences of documented elbow dislocations and concluded that valgus stress on the fully extended elbow caused the dislocation in the majority of cases [23]. This would suggest that in these cases it primarily leads to a rupture of the medial ligamentous apparatus. In 2016, Schnetzke et al. evaluated joint stability of 118 patients with ligamentous elbow dislocations [18]. All patients underwent a stability test under fluoroscopy after closed reduction. The authors reported that 36% of the patients had a higher degree of joint gap widening on the medial side with stable conditions on the lateral side.

These findings suggest that there might be a dislocation mechanism opposite to the “Horii circle of soft tissue injury”. Proof of this theory would be a near-accident MRI of a ligamentous elbow dislocation with isolated medial injury pattern. Previous MRI studies are limited in terms of inclusion criteria (MRI > 4 weeks post-trauma) and number of patients (*n* = 17 or less) studied [12, 16, 22].

Therefore, the primary aim of the present study was to analyze the soft tissue injury pattern after ligamentous elbow through early MRI investigation of a large number of patients with narrowly defined criteria. The postulated primary study hypothesis assumed existence of an alternative dislocation mechanism proceeding in the opposite direction to O'Driscoll’s Horii circle with soft tissue injury extending from medial to lateral. Secondary study objectives were the analysis of the dislocation direction and the influence of the secondary stabilizers (common flexor and common flexor origin) on joint congruence.

## Materials and methods

This multicentric retrospective case series was performed at three study centers. All consecutive patients were included in the period from March 2010 to November 2018 as long as they met the following inclusion criteria: (1) minimum age of 18 years, (2) ligamentous elbow dislocation, (3) low-energy trauma according to the definition of Mackey et al. [13] (this defined low-energy trauma as all injuries due to falls from a standing or lower height), (4) radiologically verified elbow dislocation, and (5) magnetic resonance imaging (MRI) of the affected elbow up to a maximum of 21 days post-trauma. Exclusion criteria were a high-energy trauma according to Mackey et al. [13] (e.g. car accident, motorbike accident), since in these cases there is no classical trauma mechanism with a fall onto the outstretched hand with force being applied to the elbow. Furthermore, all patients with incomplete images were excluded. After applying the inclusion and exclusion criteria, a total of 64 patients was included at the three participating study sites. The local ethics committee approved this study (837.084.14[9323-F]).

### Treatment protocol

In the participating study centers, standardized primary treatment and diagnostics were performed after ligamentous elbow dislocation. Primarily, radiographs in two planes (anteroposterior and lateral) were taken in case of clinical suspicion of elbow dislocation. After radiologically confirmed elbow dislocation, closed reduction of the elbow was performed under general anesthesia with subsequent stability testing under fluoroscopy. The stability test was not documented as standard in all cases, which is why the results of the stability test could not be included in the evaluation of this study. Following the reduction, the joint was immobilized in the upper arm cast and a timely MRI examination was performed. The MRI examination was routinely performed after ligamentous elbow dislocation within three weeks after injury, if there were no contraindications (e.g. claustrophobia, cardiac pacemaker).

### Radiographic technique and MRI protocols

Standard radiographs of the elbow in two planes (anteroposterior and lateral) were taken primarily to confirm the diagnosis of ligamentous elbow dislocation and after closed reduction. For the MRI examination, the arm was taken out of the upper arm cast and the examination was carried out in a position of the elbow that was as close as possible to full extension. All MRIs were performed using a 1.5 T scanner with dedicated elbow specific surface coils. In each case, coronal, axial, and sagittal images were available in non-fat-saturated T1-weighted and proton density-weighted sequences as well as fat-saturated T2/proton density-weighted or short tau inversion recovery (STIR) sequences. Special MRI reconstructions, such as coronal oblique images, were not performed.

### Evaluation of radiographs and MRI scans

The radiographs and MRI scans were examined independently by two observers (MS and TG). In addition, the evaluation of one observer (MS) was repeated after an interval of two weeks, and an anonymized mixed sequence of examinations was generated to prevent recall bias. The evaluators had access to the complete examinations, with the full sets of images. Radiographs and MRIs were evaluated on a medical viewing monitor with adjustable brightness and contrast control.

**Fig. 1:**
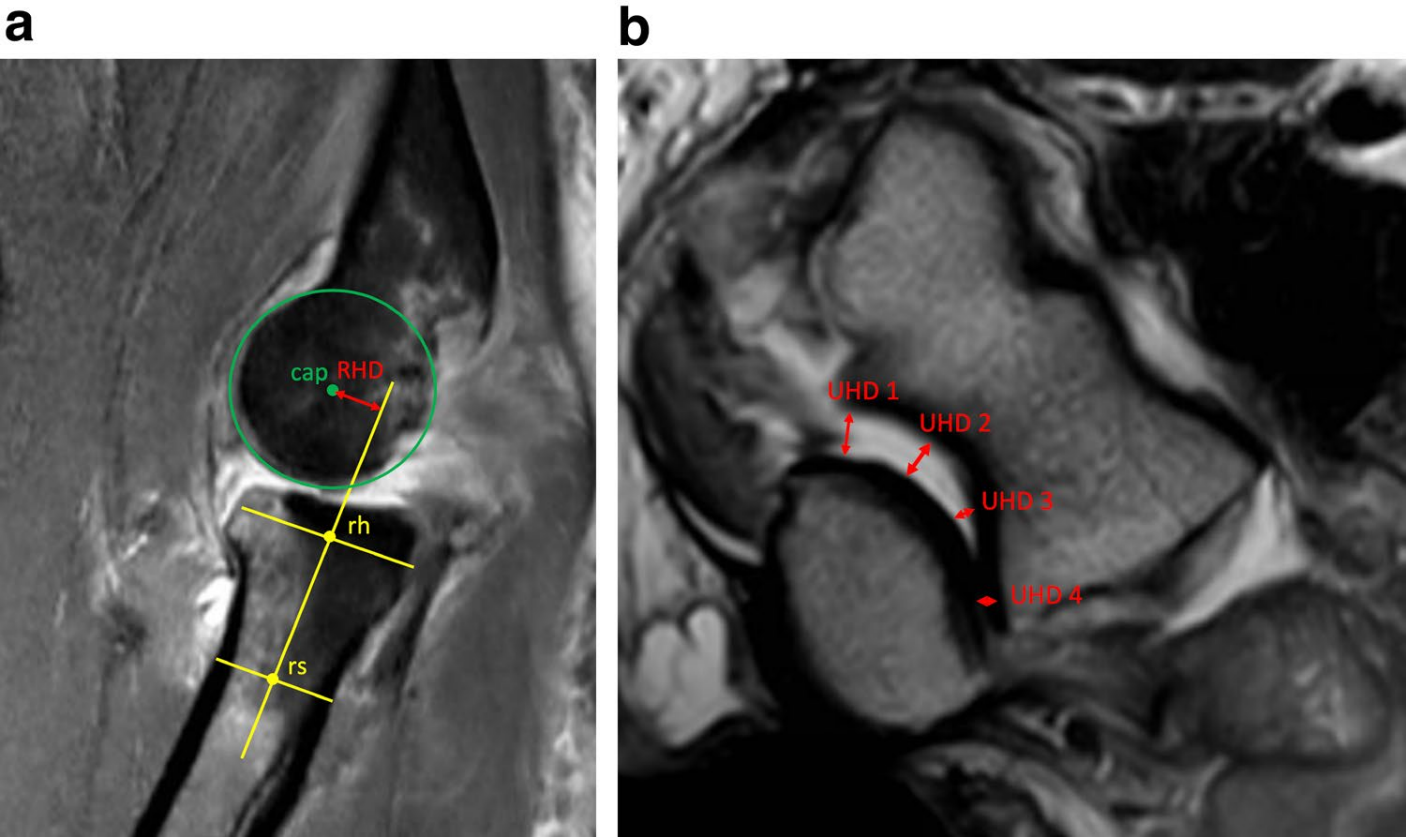
**a** Measurement of radio-capitellar incongruity: sagittal view through the center of the radial head. The rotational center of the capitulum (*cap*) was marked. The shaft axis of the radius (yellow line) is formed by the connection of *rs* (center of the radius shaft 1 cm above the tuberosity radii) and *rh* (center of the radius head). The radial head was centered on a coronal (mediolateral) and sagittal (anteroposterior) view. The distance between *cap* and the yellow line (perpendicular to the yellow line) represents the radio-humeral distance (RHD). RHD ≥ 3.4 mm was defined as the presence of radio-capitellar incongruity. **b** Measurement of axial ulno-humeral incongruity: axial view through the motion axis of the distal humerus. The ulno-humeral distance (UHD) of the trochlear joint surface to the corresponding joint surface of the olecranon was measured at the radial edge (UHD1) and ulnar edge (UHD4), and at 2 points in between (UHD2 and UHD3). For the value UHD, the difference of the lowest and highest values UHD1 to UHD4 was calculated. UHD ≥ 1.5 mm was defined as the presence of ulno-humeral incongruity

The direction of dislocation (posterolateral, dorsal, posteromedial) was determined using conventional radiographs. The MRI data sets were assessed for the presence of signal abnormalities in the area of the lateral (LCLC) and medial collateral ligament (MCL), the anterior capsule (AC), the common extensor origin (CEO), and the common flexor origin (CFO). Partial and complete ruptures as well as bone marrow edema in the attachment area of the respective ligament structures were defined as signal abnormalities. In addition, joint congruity was assessed, and joint incongruity was defined according to the MRI criteria that have been established by Hackl et al. in a previous study [7]. A radio-humeral distance greater than 3.4 mm and/or an ulno-humeral distance greater than 1.5 mm were defined as presence of joint incongruence. The measurement methods for both, the radio-humeral and the ulno-humeral distance are illustrated in Fig. 1. Furthermore, the distribution pattern of bone marrow edema was characterized by localization (no edema, lateral, medial, diffuse).

### Statistics

Descriptive statistics (mean, range, absolute, and relative frequencies) are reported for the characterization of the study population. To assess the strength of agreement between the two observers, the interobserver agreement and the agreement of one and the same observer, the intraobserver agreement, were determined for the direction of elbow dislocation based on radiographs and for the injury pattern based on MRI. Agreement strength was inferred from kappa index values in accordance with the recommendations of Landis and Koch [10]. After the analysis of the agreement strength, the cases with disagreement were discussed and a consensus was reached. Statistical analysis was performed using SPSS version 23.0.

## Results

### Epidemiology

The data sets of 64 patients with a mean age of 44 years (range 18–77 years) were analyzed. 32 patients (50%) were male and 32 were female (50%), and the left elbow was affected in 39 cases (61%) and the right in 25 cases (39%). The dominant arm was affected in 23 cases (36%). The mean time from the day of the accident to the performance of the MRI scan was 6.2 days (range 0–21 days).

### Radiological analysis

Analysis of the dislocation direction revealed that there was a posterolateral dislocation in 34 patients (53%), a posterior dislocation in 26 patients (41%), and a posteromedial dislocation in 4 patients (6%). Evaluation of the MRI images showed that there was an injury of the LCLC and AC in all 64 patients (100%). The MCL was affected in 58 patients (91%). The common extensor origin had signal abnormalities in 25 patients (39%) and common flexor origin in 20 patients (31%), respectively. Detailed analysis revealed that all the structures examined (LCLC, AC, MCL, CEO, CFO) had signal abnormalities in 7 patients (11%), 6 of whom had a posterolateral dislocation and one a posterior dislocation.

**Fig. 2:**
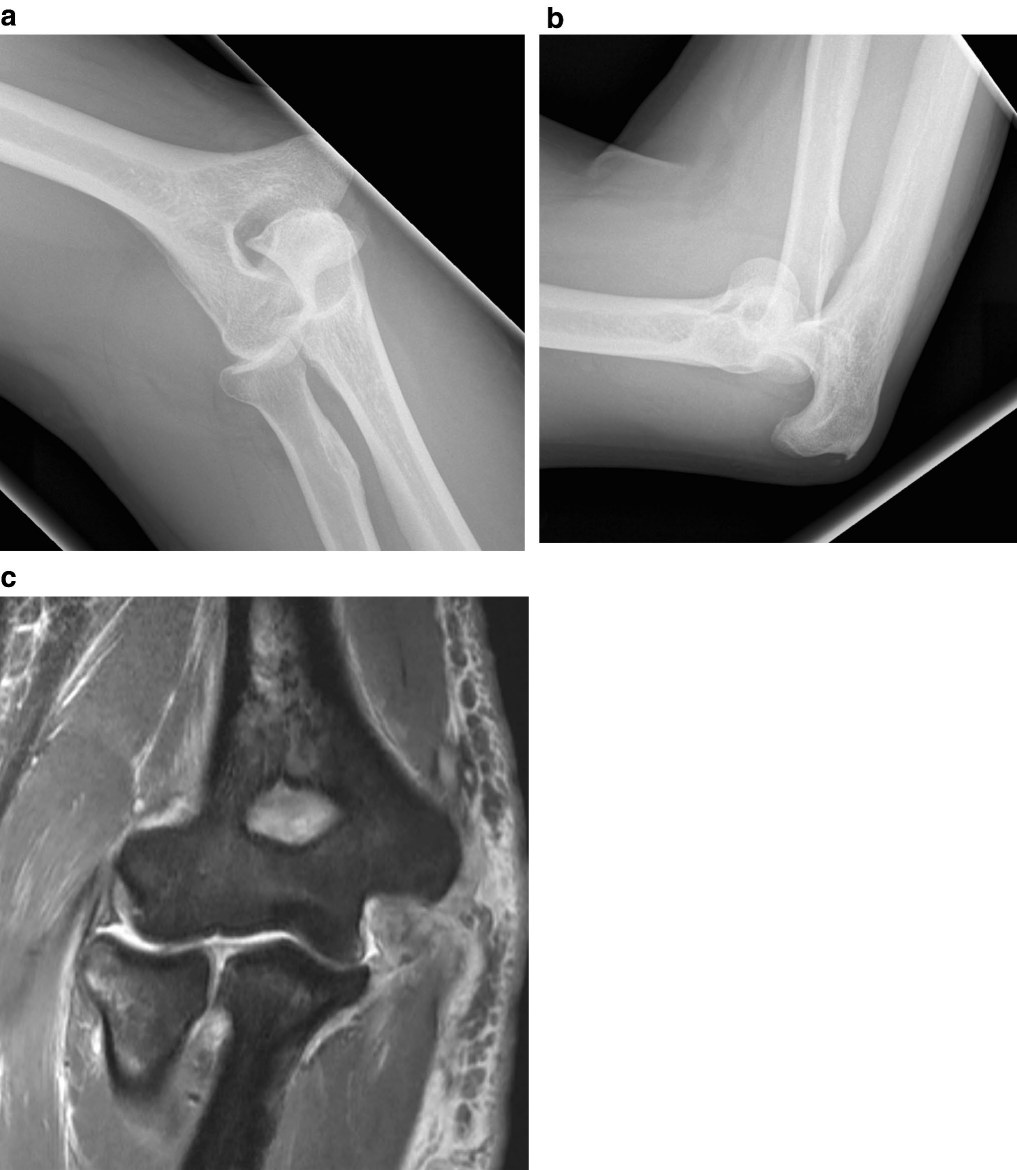
**a**, **b** Anteroposterior and lateral radiograph of a posterolateral elbow dislocation of a 39-year old patient after simple fall from standing height. **c** MRI was performed 7 days after injury. Coronal short tau inversion recovery (STIR) sequence shows signal abnormalities of the MCL and CFO (complete tear) as well as severe bone marrow edema laterally. The LCLC is partially detached from its humeral origin, whereas CEO are intact

**Fig. 3:**
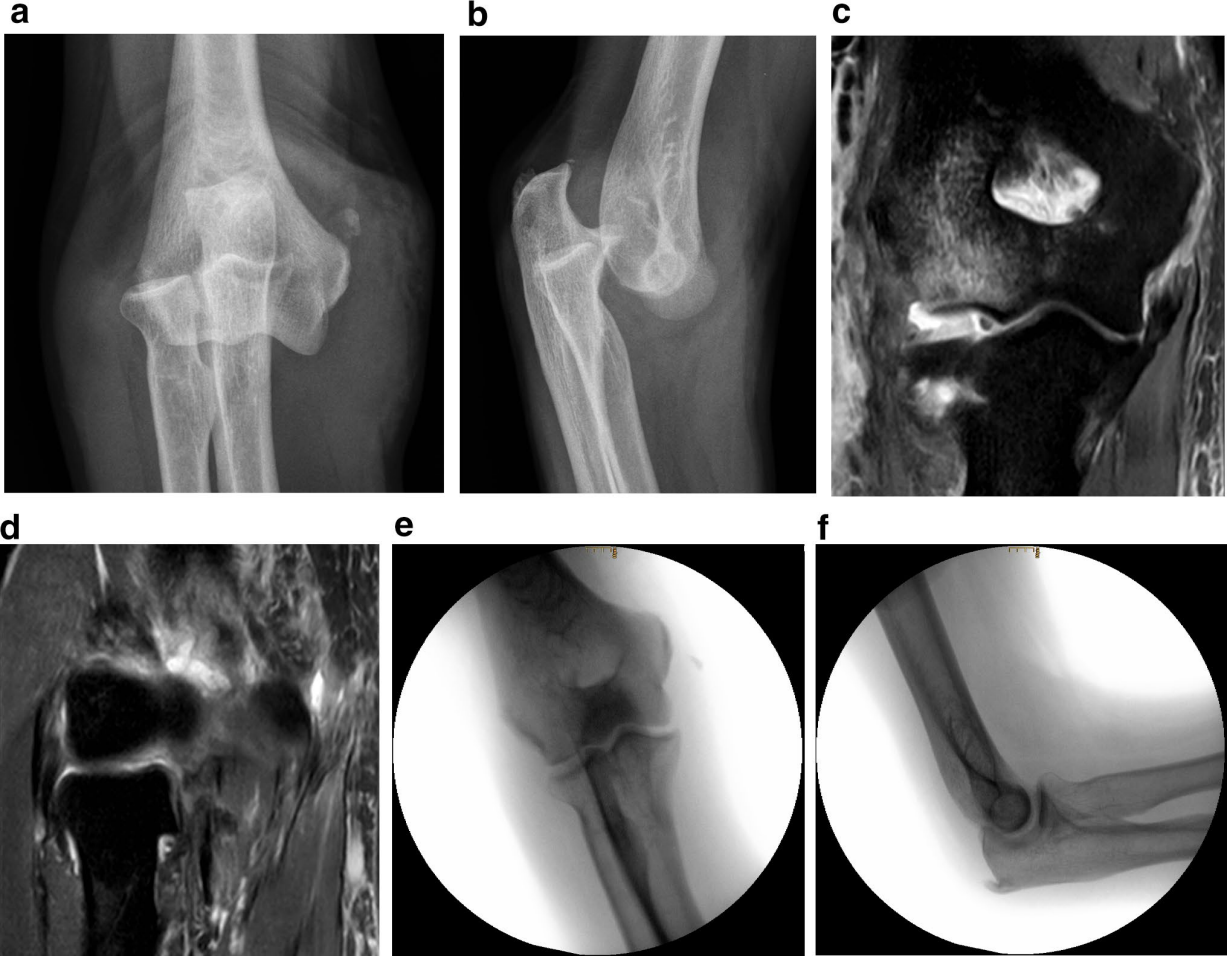
**a**, **b** Anteroposterior and lateral radiograph of a posterior elbow dislocation of a 51-year old patient after simple fall from standing height. **c** Coronal short tau inversion recovery (STIR) sequence shows signal abnormalities of the MCL and CFO (complete tear) as well as severe bone marrow edema laterally. **d** The LCLC is partially detached from its humeral origin, whereas CEO are intact. The functional integrity of the LCLC and CEO is confirmed by stress test under dynamic fluoroscopy: **e** no joint gapping during varus stress and **f** centered joint in the lateral projection under fluoroscopy

**Fig. 4:**
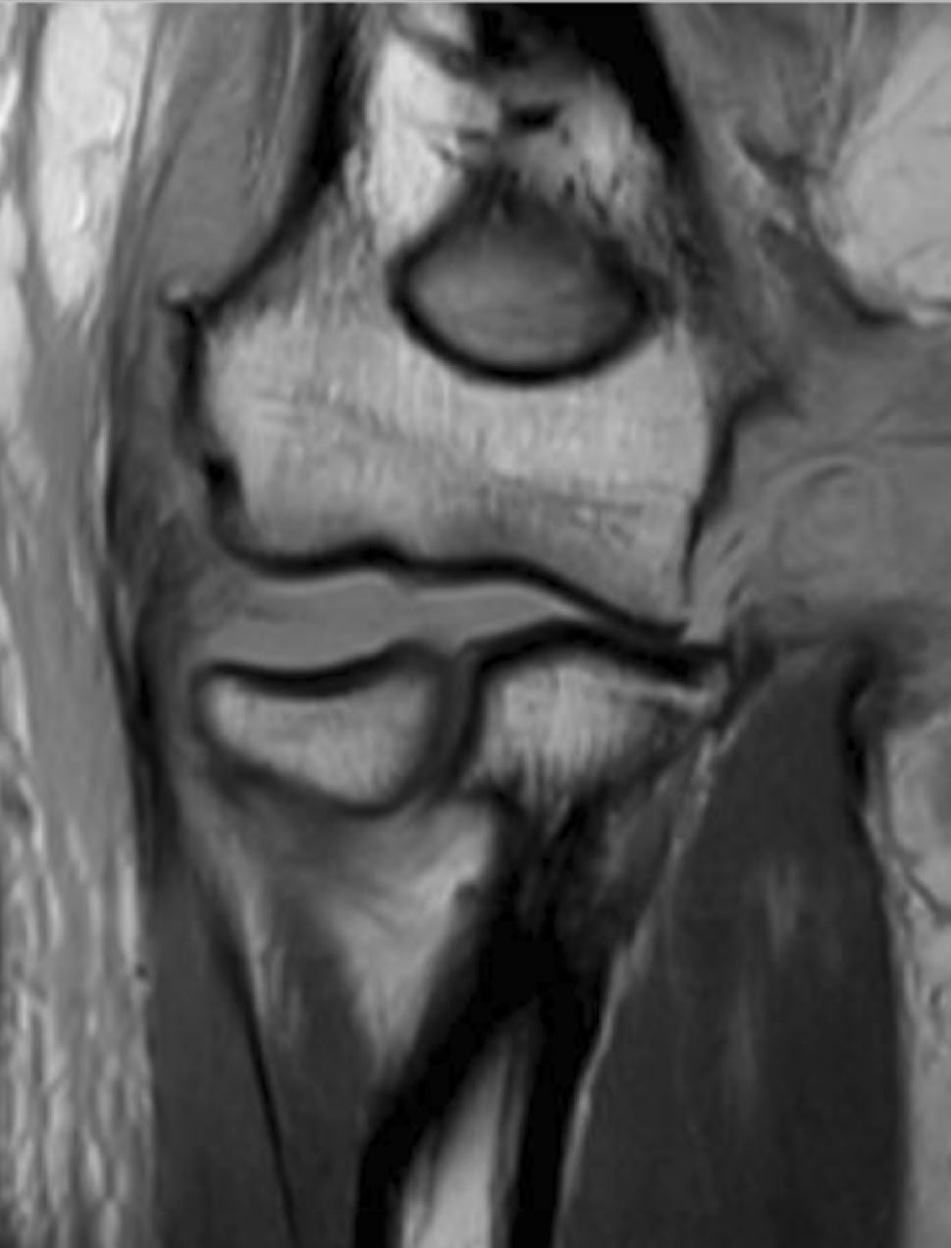
Coronal non-fat-saturated T1-weighted image: signal abnormalities of CEO, CFO, LCLC and MCL (complete tear) with marked joint incongruity

In 3 patients (*n* = 2 posterolateral, *n* = 1 posterior), isolated injury to the LCLC and AC was present. The injury pattern was greater medially than laterally (MCL, CFO, LCLC) in 11 patients (17%), 9 of whom had a posterolateral and 2 a posterior dislocation. All 11 patients with a primarily medial injury pattern exhibited marked bone marrow edema laterally in the area of the capitellum and/or radial head. In the other 53 dislocations, the distribution pattern of bone marrow edema varied considerably (no edema: 8, lateral: 30, medial:7, diffuse: 8). Detailed analysis of the LCLC of these 11 patients with greater injury pattern on the medial side revealed that the LCLC was classified as complete tear in 9 patients and as partial tear in 2 patients (Figs. 2 and 3). A detailed overview of the injury pattern classified by dislocation direction is given in Table 1. Overall, 12 patients (19%) had a joint incongruence according to the defined criteria. All 12 patients had radio-capitellar incongruence, the average radio-humeral distance was 7.8 ± 2.3 mm. 5 of these 12 patients had an additional ulno-humeral incongruence with an ulno-humeral distance of 2.7 ± 0.8 mm. Joint incongruity was present in one third of the patients (12 of 36 patients) with involvement of the extensor and/or common flexor origin, whereas there was no joint incongruity in any patients (0 of 28 patients) with intact extensor and common flexor origin (Fig. 4).

**Table 1:** Detailed injury pattern presented separately by dislocation direction

*n* (%)	Posterolatera (*n* = 34)	Posterior (*n* = 26)	Posteromedial (*n* = 4)	Total (*n* = 64)
LCLC	34 (100%)	26 (100%)	4 (100%)	64 (100%)
CEO	16 (47%)	7 (27%)	2 (50%)	25 (39%)
AC	34 (100%)	26 (100%)	4 (100%)	64 (100%)
MCL	29 (85%)	25 (96%)	4 (100%)	58 (91%)
CFO	16 (47%)	4 (15%)	0	20 (31%)

*LCLC* lateral collateral ligament complex, *CEO* common extensor origin, *AC* anterior capsule, *MCL* medial collateral ligament, *CFO* common flexor origin

### Agreement analysis

Interobserver and intraobserver agreement in terms of the dislocation direction based on radiographs was good (*k* = 0.744) or very good (*k* = 0.846). Likewise, interobserver and intraobserver agreement in the assessment of the injury pattern on MRI was good (*k* = 0.753 and 0.778, respectively).

## Discussion

In this study, all patients with ligamentous elbow dislocation exhibited a very varied and broad spectrum of soft tissue injury patterns. An isolated medial injury pattern was not found. However, a predominantly medial injury pattern was found in 11 patients (17%) with involvement of the MCL, CFO, and LCLC. All these patients exhibited marked bone marrow edema in the area of the capitellum and/or radial head. Detailed analysis revealed that 2 of these patients had only a partial tear of the LCLC (cases in Figs. 2 and 3). MRI findings in these 2 cases were consistent with a stable lateral elbow joint without joint gap widening while applying varus stress. The results of this study clearly indicate the existence of an alternative dislocation mechanism with medial force induction (pure valgus mechanism). It can be assumed that in these rare cases the soft tissue injury originates and continues from medial to anterior and thus leads to an elbow dislocation. Based on these results, the primary study hypothesis is confirmed. This alternative mechanism could be called “reversed Horii circle”.

In the literature, this combination of injuries with an isolated valgus mechanism has previously only been described without documented elbow dislocation. Cho et al. reported 7 patients with acute gross valgus instability without elbow dislocation who suffered from complete disruption of the MCL and common flexor origin, with variable degrees of tears of the anterior capsule and bone contusion of the radial head and capitellum [4]. Richard et al. reported complete avulsion of the MCL from its humeral footprint and disruption of the common flexor origin leading to acute traumatic valgus instability in 11 collegiate athletes [17]. These patients also exhibited no elbow dislocation.

Since O'Driscoll’s description of the classic dislocation mechanism, several studies have postulated a contrary dislocation mechanism to the Horii circle. In a retrospective study by Schreiber et al., the MRI scans of 16 patients with elbow dislocation were analyzed [22]. The authors describe the presence of complete rupture of the MCL with an intact lateral ulnar collateral ligament in a small proportion of patients. Based on these findings, the authors assume that there must be an alternative injury mechanism to that of O'Driscoll et al., in which dislocation of the elbow can initially also start with rupture of the MCL. The study by Schreiber et al., however, has several limitations: in addition to the small number of patients included, MRI scans up to > 50 days after the accident were included and analyzed. However, a conclusion about the injury mechanism after a period of > 4 weeks based on MRI scans is only possible to a limited extent. Furthermore, the integrity of the lateral ligament apparatus was analyzed in 4 stages (intact, low-grade partial tear, high-grade partial tear, and complete tear) and a single investigator performed the analysis without evaluating inter- or intraobserver agreement. In a previous study, we were able to show that differentiation between partial rupture of complete rupture is difficult even for experienced musculoskeletal radiologists [21].

In a further study by Schreiber et al., the authors analyzed the dislocation mechanism of 62 elbow dislocations based on documented videos on Youtube.com [23]. The authors reported that the most common mechanism appeared to involve a valgus moment to an extended elbow, which suggests a requisite disruption of the medial collateral ligament. These findings suggest that some acute elbow dislocations may result from pure valgus mechanism and therefore are distinct in nature and mechanism from posterolateral rotatory instability. However, it must be noted that this study exhibits the major limitation that an analysis of 2-dimensional data sets was performed to reconstruct a complex 3-dimensional process.

Luokkala et al. recently published a further MRI study with 16 patients and similarly to the present study analyzed the injury pattern following confirmed ligamentous elbow dislocation [12]. In this study, most patients (12/16) had a posterolateral, 3 patients a posterior, and 1 patient a posteromedial dislocation. In 15 patients, a partial or complete lesion of the LCLC was described, whereas an intact LCLC with a partially ruptured MCL was present in one patient according to the analysis. Based on biomechanical considerations, this combination of injuries would not be conceivable without a simultaneous fracture of the coronoid process. In the published diagram for this case, a bone bruise at least in the area of the humeral attachment of the LCLC is visible, which casts doubt on the integrity of the LCLC. According to the criteria of the present study, the LCLC would be classified as “signal abnormality” based on the published figure. Because of the heterogeneous injury pattern of the 16 patients studied, the authors concluded that no single mechanism-related soft tissue injury pattern of ligamentous elbow dislocation was observed, and different grades of soft tissue injury exist. Essentially, this observation coincides with the results of our study. Whereas in 3 patients (5%) only the LCLC and AC had signal abnormalities, 7 patients exhibited a marked injury pattern with involvement of the LCLC, MCL, AC, CFO, and CEO. The injury pattern cannot be deduced from the dislocation direction on the radiograph. Only a trend can be deduced, whereby the MCL may remain intact in posterolateral dislocation, whereas the considerably less common posteromedial dislocation is associated with an injury of the MCL in all cases.

Even if it was not the primary study aim, we were able to show that joint incongruity on MRI as a sign of instability in which muscular compensation is not possible was present only in patients with involvement of the extensor and/or common flexor origin. Joint incongruity was seen in 12 patients (19%), with involvement of the common extensor and/or flexor origin in all of these 12 patients. Joint incongruity following dislocation is known to be a warning sign of chronic instability [7, 18]. The secondary stabilizers (common extensor and flexor origin) therefore appear to play a determining role in the stability of the elbow joint. These results are in line with previous studies that have investigated the important role of the flexor-pronator muscles as active stabilizers against valgus stress [11, 24].

## Limitations and strengths

This study has several limitations. A power analysis and a sample size calculation have not been performed. However, due to a lack of literature data, a sample size calculation was not possible in this case series. MRI findings were not compared with intraoperative findings. The difficulties in assessing ligament injuries with MRI after elbow dislocations is well-known [21]. We know from earlier studies that partial tears might be detected with low sensitivity, and the structures that contribute significantly to stability may be poorly visualized in MRI, such as the lateral ulnar collateral ligament [2, 3, 8, 14]. Lesions might have been underestimated on MRI and were not verified e.g. with instability testing or intraoperative findings. A stability test was only available for some of the patients in the present study (e.g. patient example in Fig. 3) and was therefore not included in the evaluation. Therefore, the described findings in this study probably do not show the full picture of the injuries. In addition, only good interobserver agreement regarding the dislocation direction based on radiographs was found. In some cases, a clear distinction between direct posterior and posterolateral or posteromedial dislocation was not possible with certainty.

The strengths of this study are that it was a large series of patients, and the initial dislocation direction could be recorded in all patients. MRI scans were performed in all patients within 3 weeks after the initial injury. Two experienced orthopedic surgeons evaluated the data independently, with good interobserver reliability.

## Conclusion

Ligamentous elbow dislocation results in a heterogeneous spectrum of ligamentous and muscular soft tissue injury. A small proportion of patients clearly showed medially pronounced injury patterns. These findings strongly indicate existence of a “reversed Horii circle” with an underlying valgus mechanism with medial force induction originating and continuing from medially to anteriorly.
